# Adjudication of etiology of acute kidney injury: experience from the TRIBE-AKI multi-center study

**DOI:** 10.1186/1471-2369-15-105

**Published:** 2014-07-04

**Authors:** Jay L Koyner, Amit X Garg, Heather Thiessen-Philbrook, Steven G Coca, Lloyd G Cantley, Aldo Peixoto, Cary S Passik, Kwangik Hong, Chirag R Parikh

**Affiliations:** 1 Department of Internal Medicine, Program of Applied Translational Research, Yale University and Veterans Affairs Medical Center, 60 Temple Street, Suite 6C, New Haven, CT 06510, USA; 2 Section of Nephrology, Department of Medicine, University of Chicago, Pritzker School of Medicine, Chicago, IL, USA; 3 Department of Cardiothoracic Surgery, Danbury Hospital, Danbury, CT, USA; 4 Division of Nephrology, Departments of Medicine, Epidemiology and Biostatistics, University of Western Ontario, London, ON, Canada

**Keywords:** Acute kidney injury, Acute tubular necrosis, Cardio-thoracic surgery, Adjudication

## Abstract

**Background:**

Adjudication of patient outcomes is a common practice in medical research and clinical trials. However minimal data exists on the adjudication process in the setting of Acute Kidney Injury (AKI) as well as the ability to judge different etiologies (e.g. Acute Tubular Necrosis (ATN), Pre-renal Azotemia (PRA)).

**Methods:**

We enrolled 475 consecutive patients undergoing cardiac surgery at four sites of the Translational Research Investigating Biomarker Endpoints in AKI (TRIBE-AKI) study. Three expert nephrologists performed independent chart review, utilizing clinical variables and retrospective case report forms with pre intra and post-operative data, and then adjudicated all cases of AKI (n = 67). AKI was defined as a > 50% increase in serum creatinine for baseline (RIFLE Risk). We examined the patterns of AKI diagnoses made by the adjudication panel as well as association of these diagnoses with pre and postoperative kidney injury biomarkers.

**Results:**

There was poor agreement across the panel of reviewers with their adjudicated diagnoses being independent of each other (Fleiss’ Kappa = 0.046). Based on the agreement of the two out of three reviewers, ATN was the adjudicated diagnosis in 41 cases (61%) while PRA occurred in 13 (19%). Neither serum creatinine or any other biomarker of AKI (urine or serum), was associated with an adjudicated diagnosis of ATN within the first 24 post-operative hours.

**Conclusion:**

The etiology of AKI after cardiac surgery is probably multi-factorial and pure forms of AKI etiologies, such as ATN and PRA may not exist. Biomarkers did not appear to correlate with the adjudicated etiology of AKI; however the lack of agreement among the adjudicators impacted these results.

**Trial registration:**

Clinicaltrials.gov: NCT00774137

## Background

Acute Kidney Injury (AKI), regardless of the definition employed, is a common complication following adult cardiac surgery [1,2]. Increasing acceptance of standardized definitions of AKI amongst nephrologists and intensivists has advanced several aspects of AKI research yet the practical issue of what constitutes “true AKI” clinically, remains unsettled [3-8]. Novel biomarkers have demonstrated promising associations with AKI [3,4], leading to a resurgence in clinical trials in the setting of AKI [9]. However, given the suboptimal performance of some biomarkers, there is only limited data to support the role of accurate adjudication of AKI to improve the diagnostic and discriminative performance of traditional and modern markers of renal function [10].

Recent research has focused on augmenting serum creatinine’s limited ability to detect true renal tubular injury, rather than a temporary change in glomerular filtration (pre-renal azotemia, PRA) [11-15]. Conceptually, PRA, defined as a transient decrease in the effective perfusion of the kidney, either from volume depletion or relative hypotension, should spare damage to the kidney/renal tubules in comparison to other intrinsic (intra-renal) causes of kidney injury [16,17]. Some maintain that attempting to clinically distinguish PRA from intrinsic kidney injury is not feasible and likely unnecessary as all AKI lies on a spectrum [18]. A limited number of non-cardiac surgery studies have had varying success in attempting to adjudicate AKI events and tease out the ability of novel biomarkers to distinguish PRA from direct tubular injury [10,14,15].

Separating PRA from intrinsic forms of AKI, including its most severe form acute tubular necrosis (ATN), is crucial as future clinical trials will be designed and powered to treat those with evidence of cellular kidney death (ATN). Thus recruiting, enrolling and studying patients with ATN is of the utmost importance, ensuring that we are treating a cohort of patients at the highest risk for the most severe forms of AKI and the worst clinical outcomes. Enrolling patients who have PRA, and who will not develop ATN will not only, increase study costs and weaken findings but also potentially expose patients to a therapeutic that they will not benefit from and thus increase patient risk. Pragmatically, all AKI (PRA, ATN, obstruction, athero-embolic disease and other sources) are at their simplest defined in terms of a rise in serum creatinine and/or a decrease in urine output, thus differentiation utilizing these two traditional tools is difficult. Compounding this dilemma, prolonged PRA may progress across the AKI spectrum to overt ATN over a patient specific time course. Unfortunately the pathognomonic “muddy brown casts” of ATN are not always present early in the course of AKI or at the time of renal consultation, rendering ATN a diagnostic possibility even in the absence of these clinical findings [19]. Finally, several of the other tools (fractional excretion of sodium and urea) used to distinguish between PRA and ATN have been shown to be ineffective following cardiac surgery [11,20].

In this nested prospective cohort sub-study of the Translational Research Investigating Biomarker Endpoints in AKI (TRIBE AKI) study, we established a framework for the AKI adjudication process as well as provide adjudication outcomes in adults who developed AKI [3]. Additionally, we investigated the association of several AKI biomarkers (traditional and modern) with the adjudicated AKI outcome.

## Methods

### Study sample

We conducted a prospective cohort sub-study of adults undergoing cardiac surgery (coronary artery bypass grafting [CABG], surgery for valve disease and both) at four academic medical centers in North America between July 2007 and December 2009. All patients were at high risk for AKI, defined by the presence of one or more of the following criteria: 1) Pre-existing renal impairment (baseline serum creatinine > 2 mg/dL [177 μmol/L]) 2*)* An ejection fraction <35% or grade 3 or 4 left ventricular dysfunction, 3) Age > 70 years, 4) History of diabetes mellitus, 5) Scheduled to undergo a concomitant CABG and valve surgery, or 6*)* Scheduled to undergo a repeat revascularization surgery.

Adult patients were excluded if they had any of the following: 1) evidence of AKI prior to surgery (prolonged pre-operative surgical hospitalization with 2 or more pre-operative creatinine values with a > 0.3 mg/dl (26.5 μmol/L) increase), 2) prior kidney transplantation, 3) a pre-operative serum creatinine level > 4.5 mg/dL (400 μmol/L) 4) pre-existing end-stage renal disease, 5) administration of a nephro-toxic drug preoperatively (except for IV contrast, angiotensin converting enzymes inhibitors and angiotensin II receptor blockers), 6) planned surgery for a ventricular assist device or congenital heart disease or 7) if they did not have one serum creatinine test prior to the surgery in the hospital laboratory used by the hospital where the subject will undergo their surgery.

All participants provided written informed consent and the study was approved by The Institutional Review Boards (IRB) of The University of Chicago, Yale University and Danbury Hospital and the University of Western Ontario Research Ethics Board. This clinical study was registered at Clinicaltrials.gov as NCT00774137 on October 16, 2008 and adheres to the STROBE guidelines for cohort studies (Additional file 1).

The primary outcome of AKI was determined by the daily creatinine measurements during the first five hospital days. AKI was defined by RIFLE “Risk” or higher: an increase of ≥ 50% from pre-operative baseline [5]. Additionally, clinical care, including the decision to initiate renal replacement therapy or other renal interventions, was made by the primary service and the Nephrology Consult service, without involvement of the study investigators.

Pre-operative creatinine was measured as part of routine clinical care in each hospital’s clinical lab. Estimated glomerular filtration rates (eGFR) were calculated using the modified MDRD equation [21,22]. Serum creatinine was collected daily during the hospital stay. Past medical and surgical histories as well as preoperative co-morbid conditions were obtained from the patient and the clinical record, using standardized definitions of the Society of Thoracic Surgeons (STS) data collection tool. These included, but were not limited to variables that had been included in the prior STS registry risk-assessment tool for predicting AKI after cardiac surgery, as well as others related to kidney function estimation. These included demographics (age, sex, race), co-morbidities (hypertension, diabetes mellitus, heart failure, prior myocardial infarction), preoperative medication utilization and surgical characteristics (elective or urgent; bypass, valvular surgery, or both; prior cardiac operation). Patients requiring emergent surgery were excluded from this study. Additionally, intra-operative variables (cardio pulmonary bypass time, aortic cross clamp time, nephro-toxins and utilization of cardioplegia, intra-aortic balloon pumps, blood products, and vaso-active medications) were also collected. Finally post-operative factors including but not limited to daily serum creatinine, daily urine output and fluid balance, post-operative medication utilization (including detailed diuretic and vaso-active medication records), post-operative complications (prolonged intubation, re-operation, need for blood products, length of ICU and hospital stay, modality and timing of any RRT and mortality data) were all recorded.

### Outcome definitions

**Figure 1:**
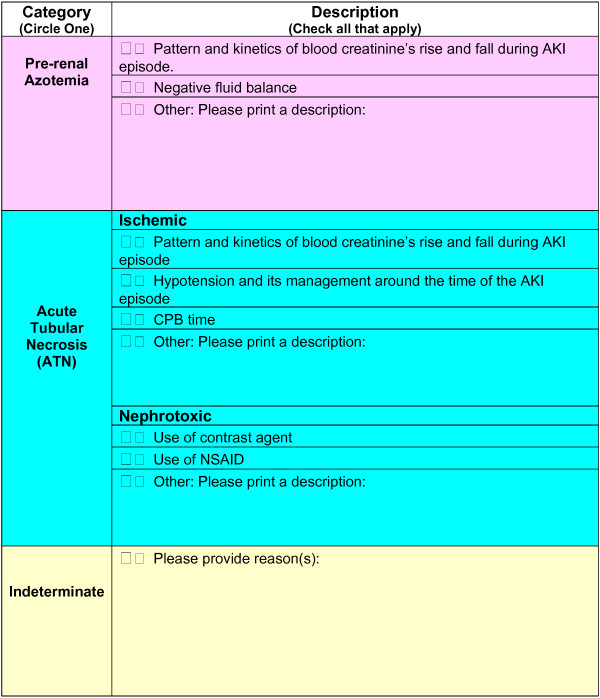
**Blank adjudication form – This figure demonstrates the blank form that individual panelists completed after their chart review for those patients with AKI in the post-operative period.** Adjudicators we asked to supply their rationale for selecting the etiology of AKI (PRA, ATN or Indeterminate).

All patients who developed post-operative AKI had their entire case report form and hospital course independently retrospectively reviewed by a panel of 3 independent academic nephrologists (SCG; LGC, AP). The committee members were chosen on the basis of expertise in clinical nephrology and represented individuals across a spectrum of years in practice, and from federal and academic hospital settings. None of the adjudicators were involved with clinical care of the patients in this study.Retrospectively the adjudication panel individually utilized de-identified case report forms, which contain all of the aforementioned pre-, intra and post-operative data to assign an AKI diagnosis of ATN, PRA or indeterminate. Additionally, all 3 panelists were asked to complete a checklist following their case-decision providing the rationale behind their diagnostic decision (Figure 1). In those cases that were indeterminate, the panelists were required to supply the rationale for their decision. The panelists were blinded to patient identifiers, the names/location of the treating physicians and all data on novel biomarkers. Finally, all panelists operated independently and were blinded to the adjudication results of their 2 co-adjudicators. If two adjudicators agreed on any etiology, then this consensus was considered to be a final diagnosis for the patient. At the end of study, there was an in-person meeting of the three adjudicators to discuss the cases where no two adjudicators agreed on a given diagnosis. Cases were discussed until two of the three adjudicators could agree on a diagnosis.

### AKI biomarker measurements

Urine IL-18 and NGAL were measured with the ARCHITECT® assay (Abbott Diagnostics, Abbott Park, IL). We measured urine creatinine by the modified Jaffe reaction. We measured plasma NGAL using the Triage NGAL immunoassay, in conjunction with the Triage Meter (Biosite Inc., San Diego, CA) CV of 10% [3]. Urine albumin was reported as the ratio to urinary creatinine (ACR) and was measured via immunoturbidimetry on a Siemens Dimension Plus with HM clinical analyzer per the manufacturer’s instructions [23]. BNP was measured via the Biosite Triage meter (Biosite, San Diego California) method [24]. KIM-1 LFABP and urine Cystatin C were all measured via Sekisui Diagnostics LLC developed antibody ELISA assays [25,26].

### Statistical considerations

Continuous variables were compared with two-sample *t*-test or Wilcoxon rank sum test and dichotomous variables with the chi-square test or Fisher’s exact test. Fleiss’ kappa was used to determine inter-rater independence across all 3 adjudicators while Cohen’s kappa was used for pairs of adjudicators.

## Results

We enrolled 478 of patients in this sub-study Three subjects were excluded due to violations of inclusion/exclusion criteria, leaving 475 subjects in the final analyses. Sixty-seven (14%) of these individuals developed AKI, defined by RIFLE “Risk”. Of those individuals who developed AKI, 6 (1.2% of the total, 9% of those with AKI) went on to require RRT. Of the 67 subjects with AKI, there was 100% agreement amongst the 3 adjudication panelists on only 13 cases (19%). In 11 (16%) subjects, there was complete agreement that ischemic ATN was the cause of AKI; while PRA was the adjudicated diagnosis in the remaining 2 cases (3.0% of the total). In none of the 67 cases did all 3 panelists agree that the cause of AKI was indeterminate (Table 1).

**Table 1:** Breakdown of diagnoses made via the AKI adjudication panel

	Final adjudication result*			**Total n (%)**
**Panelists adjudications**	**ATN**	**PRA**	**Indeterminate**	
3ATN	11			11 (16)
3PRA		2		2 (3)
2ATN; 1PRA	16			16 (24)
2ATN; 1IND	11			11 (16)
2PRA; 1ATN		4		4 (6)
2PRA; 1IND		6		6 (9)
2IND; 1ATN			2	2 (3)
2IND; 1PRA			5	5 (8)
1ATN; 1PRA; 1IND	3	1	6	10 (15)
**Total**	**41**	**13**	**13**	**67 (100)**

ATN = Acute Tubular Necrosis, PRA = Pre-Renal Azotemia.

*Final Adjudication Result was assigned if at least 2 adjudicators agreed on the AKI etiology. Adjudicators met in-person to reach a final consensus on the 10 cases where the initial adjudication differed across all 3 adjudicators.

In the 56 (81%) remaining cases of AKI there was not a unanimous consensus; however, in 46 (69% of the total) cases, 2 adjudicators agreed on a diagnosis. The most common scenario was 2 reviewers diagnosing ATN with the third diagnosing PRA (25% of the total cases); a complete list of the adjudications can be found in Table 1. There were 10 (14.9%) cases in which all 3 panelists originally disagreed about the underlying diagnosis, with each panelist choosing separate options; PRA, ATN and Indeterminate. During an in-person meeting these 10 cases were eventually judged to be 1 case of PRA, 3 cases of ATN and 6 cases of indeterminate. The clinical characteristics of the adjudicated sub-groups (ATN, PRA and Indeterminate) are depicted in Table 2. Individuals adjudicated to have ATN were more likely to have a higher peak post-operative serum creatinine, lower urine output during the first 48 post-operative hours and a longer duration of AKI compared to those with PRA. Those judged to have PRA were more likely to have pre-operative exposure to radio-contrast.

**Table 2:** Clinical characteristics of the 67 cases of AKI based on final adjudication

	**Pre-renal azotemia (n = 13)**	**Acute tubular necrosis (n = 41)**	**Indeterminate (n = 13)**	p-value*
**Pre-op Factors**				
Age (years)	72.9 (11.6)	72.5 (9.4)	75.7 (8.4)	0.68
Female sex	6 (46%)	16 (39%)	5 (39%)	0.65
BMI	30.5 (5.6)	32.3 (7.2)	29.1 (6.8)	0.51
Caucasian Race	13 (100%)	38 (93%)	11 (85%)	0.32
Diabetes Mellitus	9 (69%)	20 (49%)	4 (31%)	0.20
Baseline Serum Creatinine (mg/dL)	1.01 (0.31)	1.14 (0.38)	1.06 (0.34)	0.39
Baseline eGFR (CKD-EPI) ml/min	68.99 (18.77)	63.28 (21.95)	67.68 (21.33)	0.37
Repeat Cardiac Surgery	2 (17%)	9 (22%)	3 (23%)	0.69
Pre-op Contrast Exposure	3 (23%)	2 (4.9%)	1 (7.7%)	0.05
**Intra-op Factors**				
Type of Surgery				0.95
CABG alone	5 (39%)	15 (37%)	9 (75%)	
Valve alone	3 (23%)	11 (27%)	3 (25%)	
CABG and Valve	5 (39%)	15 (37%)	0 (0%)	
Off-Pump Surgery	0 (0%)	2 (5%)	3 (23%)	0.44
CPB Time (min)	103.54 (44.34)	139.03 (62.11)	117.44 (59.54)	0.07
Cross Clamp Time (min)	84 (40.66)	95.21 (44.08)	81.89 (47.01)	0.45
**Post op Factors**				
Length of ICU stay (days)	2.38 (1.45)	12.02 (30.74)	4.38 (4.44)	0.29
Length of Hospitalization (days)	6.77 (1.92)	17.49 (32.23)	7.85 (3.65)	0.07
Received RRT	0 (0%)	6 (15%)	0 (0%)	0.14
Maximum Serum Post op Creatinine (mg/dl)	1.64 (0.55)	2.18 (0.76)	1.84 (0.58)	0.03
Absolute Increase of Maximum Serum Creatinine (mg/dL)	0.63 (0.25)	1.04 (0.55)	0.77 (0.39)	0.01
Maximum% Increase of Serum Creatinine (%)	62 (12)	96 (59)	76 (48)	0.03
Duration of AKI (Days)	1 [1, 2]	2 [1, 4]	1 [0.5, 1]	0.0002
**Urine Output/Fluid Balance**				
Urine output 0-6 hr (mls)	1435 [1185, 2643]	1085 [890, 1395]	1015 [667, 1600]	0.04
Urine output day 2 (mls)	1916 [1151, 3200]	1030 [710, 1505]	2095 [925, 3075]	0.02
Urine output day 3 (mls)	2000 [1620, 3690]	1315 [800, 1760]	1133 [733, 1950]	0.01
Net fluid Balance 0-6 hr	1716 [665, 1961]	1488 [437, 2504]	1251 [783, 1836]	0.65
Net fluid Balance Day 2	539 [-1920, 1839]	2040 [68, 3438]	79 [-835, 2110]	0.16
Net fluid Balance Day 3	510 [-1493, 790]	225 [-386, 1532]	-122 [-572, 1054]	0.43
RIFLE “I” n (%)	0 (0%)	16 (39%)	3 (23%)	0.01
RIFLE “F” n (%)	0 (0%)	4 (9.8%)	1 (7.7%)	0.24
Hospital Mortality n (%)	0 (0%)	6 (15%)	1 (7.7%)	0.16

*P-value is the result of comparison between ATN and PRA. Wilcoxon test used for continuous variables and Chi-square Test for categorical variables.

Investigation into the diagnosis patterns of the 3 individual adjudicators demonstrated that despite receiving the same instruction set and diagnostic materials, there was tremendous variability across all 3 panelists (Table 3). The adjudications of our 3 panelists were statistically independent of each other (Fleiss’ Kappa = 0.046). However when looking at individual adjudicator pairings no pairing provided a Cohen’s Kappa < 0.05. (Adjudicator 1 and 2 Cohen kappa = 0.24; Adjudicator 1 and 3 Cohen kappa = 0.06; Adjudicator 2 and 3 Cohen kappa = 0.10).

**Table 3:** Breakdown of the panelist’s diagnoses

	**Adjudicator #1**	**Adjudicator #2**	**Adjudicator #3**
ATN, n (%)	49 (73.2)	40 (59.7)	14 (20.9)
PRA, n (%)	9 (13.4)	9 (13.4)	39 (58.2)
Indeterminate, n (%)	9 (13.4)	18 (26.9)	14 (20.9)

Table 4 demonstrates the mean pre and post-operative biomarker values throughout the first 5 days for all subjects adjudicated to either ATN or PRA. No biomarker was significantly different across these 2 groups during the first 24 post-operative hours. There was a statistical difference between KIM-1 values at Day 2 but this effect was not sustained over subsequent post-operative days. Serum Creatinine and Serum Cystatin C were not statistically different until Day 3 with both of these effects being sustained over the next 2 post-operative days.

**Table 4:** Post-operative biomarker values for patients adjudicated to PRA and ATN

**Biomarkers**	**Pre-renal azotemia (N = 13)**	**Acute tubular necrosis (N = 41)**	**p-value Wilcoxon**
**Urine NGAL**			
Pre-op	34 (41)	42 (79)	0.33
Day 1 0-6 hr	74 (169)	173 (617)	0.73
Day 1 6-12 hr	328 (978)	50 (68)	0.81
Day 1 12-18 hr	85 (108)	76 (105)	0.90
Day 2	81 (79)	95 (132)	0.77
Day 3	57 (72)	91 (112)	0.17
Day 4	110 (124)	102 (110)	0.97
Day 5	60 (72)	103 (164)	0.34
**Urine IL-18**			
Pre-op	28 (26)	63 (263.93)	0.49
Day 1 0-6 hr	68 (108)	147.13 (517.98)	0.39
Day 1 6-12 hr	249 (640)	168.01 (416.33)	0.85
Day 1 12-18 hr	77 (37)	211.72 (328.66)	0.24
Day 2	91 (55)	186.03 (298.02)	0.79
Day 3	108 (166)	103.42 (191.36)	0.72
Day 4	46 (43)	73.07 (107.88)	0.83
Day 5	25 (20)	42.79 (77.48)	0.85
**Urine KIM 1**			
Pre-op	3.8 (6.2)	3.0 (6.7)	0.73
Day 1 0-6 hr	1.7 (2.5)	1.7 (2.6)	0.57
Day 1 6-12 hr	2.7 (3)	2.4 (1.7)	0.62
Day 2	9.1 (4.9)	5.4 (4.0)	**0.01**
Day 3	14.8 (9.5)	12.4 (11.7)	0.28
Day 4	9.2 (9.0)	9.0 (9.7)	0.97
Day 5	4.5 (3.4)	6.2 (8.6)	0.96
**Urine LFABP**			
Pre-op	33 (57)	27 (86)	0.77
Day 1 0-6 hr	68 (65)	118 (156)	0.66
Day 1 6-12 hr	84 (157)	97 (141)	0.29
Day 2	63 (111)	75 (125)	0.95
Day 3	64 (116)	52 (92)	0.69
Day 4	126 (173)	98 (140)	0.71
Day 5	92 (134)	79 (126)	0.52
**Urine Cystatin C**			
Pre-op	0.24 (0.18)	0.27 (0.39)	0.54
Day 1 0-6 hr	0.20 (0.13)	0.22 (0.13)	0.81
Day 1 6-12 hr	0.27 (0.21)	0.21 (0.13)	0.40
Day 2	0.25 (0.17)	0.26 (0.22)	0.85
Day 3	1.12 (2.65)	0.42 (0.94)	0.42
Day 4	1.94 (3.46)	0.57 (1.07)	0.63
Day 5	1.63 (3.12)	1.14 (3.22)	0.77
**Urine Albumin**			
Pre-op	175 (379)	133 (287)	0.89
Day 1 0-6 hr	35 (36)	56 (109)	0.5
Day 1 6-12 hr	48 (77)	47 (55)	0.39
Day 1 12-18 hr	77 (154)	95 (143)	0.42
Day 2	72 (136)	71 (84)	0.26
Day 3	107 (168)	77 (77)	0.84
**Urine ACR**			
Pre-op	0.27 (0.74)	0.16 (0.3)	0.94
Day 1 0-6 hr	0.21 (0.19)	0.15 (0.17)	0.32
Day 1 6-12 hr	0.11 (0.18)	0.09 (0.13)	0.64
Day 1 12-18 hr	0.07 (0.1)	0.11 (0.16)	0.45
Day 2	0.06 (0.08)	0.11 (0.14)	0.13
Day 3	0.1 (0.14)	0.08 (0.09)	0.64
**Serum NGAL**			
Pre-op	82 (30)	99 (54)	0.43
Day 1	214 (91)	259 (90)	0.20
Day 2	213 (123)	236 (123)	0.54
Day 3	162 (68)	224 (132)	0.17
Day 4	102 (54)	184 (133)	**0.04**
Day 5	87 (31)	147 (86)	0.09
**Serum BNP**			
Pre-op	152 (94)	194 (226)	0.67
Day 1	111 (118)	112 (118)	0.67
Day 2	297 (202)	337 (224)	0.50
Day 3	364 (180)	373 (219)	0.76
Day 4	525 (151)	442 (297)	0.11
Day 5	458 (209)	431 (208)	0.67
**Serum Creatinine**			
Pre-op	0.99 (0.32)	1.14 (0.39)	0.31
Day 1	1.15 (0.45)	1.32 (0.46)	0.26
Day 2	1.53 (0.56)	1.68 (0.67)	0.5
Day 3	1.39 (0.57)	2.06 (0.72)	**<0.01**
Day 4	1.21 (0.4)	1.88 (0.67)	**<0.01**
Day 5	1.1 (0.33)	1.66 (0.58)	**<0.01**
**Serum Cystatin C**			
Pre-op	0.92 (0.29)	1.13 (0.4)	0.13
Day 1	0.86 (0.27)	1.02 (0.31)	0.12
Day 2	1.04 (0.47)	1.26 (0.43)	0.11
Day 3	1.08 (0.34)	1.54 (0.52)	**<0.01**
Day 4	0.99 (0.26)	1.62 (0.52)	**<0.01**
Day 5	1.02 (0.27)	1.49 (0.51)	**0.01**

(All values Mean (SD)).

## Discussion

The development of AKI following cardiac surgery is extremely complex. Preoperative factors such as exposure to nephro-toxins (e.g. radio contrast from cardiac catheterization) and pre-existing conditions (congestive heart failure, diabetes mellitus and chronic obstructive lung disease) contribute and interact with operative factors such as emergent surgery, intra-operative hypotension and cardio-pulmonary bypass (CPB) pump time in determining AKI and patient outcomes. The interplay of these factors along with the patient’s post-operative course (e.g. mechanical ventilation, use of vaso-active medications, balloon pumps or diuretics) all may contribute to the development of AKI. As such determining the etiology of AKI while relying on the clinical course and changes in serum creatinine and urine output is therefore often a subjective and challenging task. As AKI research moves into an era of clinical trials, it will be increasingly important to ensure that all subjects enrolled with AKI have a similar etiology of renal dysfunction as interventions aimed at ischemia-induced ATN may not work in nephrotoxin or contrast related injury, let alone in the setting of functional PRA. Just as previous interventions have failed, in part, because they were initiated too late, future agents may not work if they are investigated across a broad spectrum of AKI [27]. Our initial experiences with the adjudication of cardiac surgery associated AKI demonstrate that while feasible, this process is not perfect.

It is extremely important that clinical definitions of AKI are harmonized beyond a simple elevation in serum creatinine or decrease in urine output. Our experience demonstrates that not all AKI following cardiac surgery is the same and that a clean, precise, one-hit phenotype likely does not exist. In the past cardiac surgery associated AKI was thought to be a homogenous clinical entity predominantly due to renal ischemia-reperfusion injury however over the last decade investigators have recognized that it is a multi-factorial process that involves ischemia-reperfusion, inflammation, variation systemic hemodynamics, athero-embolic disease and nephrotoxins [28,29]. With so many factors to consider it is not surprising that there was a large degree of variation in the adjudicated diagnoses across our 3 panelists with all 3 adjudicators agreeing only 19% of the time. Their discrepant clinical decision making when possessing identical information is readily apparent in Table 3. In the end, 41 (61%) subjects were judged to have ATN while 13 (19%) had PRA with several of these cases requiring an in-person discussion in order to arrive at a final diagnosis. It is important to reiterate that the evaluation process was done retrospectively months after the hospitalization and that all three reviewers had access to the same clinical information, but not the novel biomarkers data. Thus, at most, only 60% of AKI subjects would have been appropriate for enrollment in a therapeutic study to treat or diminish the effects of ATN. This is an important issue, as future trials will seek to identify cohorts in the early post-operative period who are at risk for ATN while attempting to minimize the enrollment of those with functional forms of AKI. In our study, differences in glomerular filtration markers (Serum Creatinine and Cystatin C) were not apparent until post-operative day 3 (Table 4). Such a timeframe is likely outside of the therapeutic window for many novel treatments that require early access to the injured nephron. Relying solely on serum creatinine kinetics therefore is not a viable option for achieving diagnostic distinction.

Not surprisingly, given the poor agreement across our 3 adjudicators, no biomarker, consistently distinguished etiology of AKI within the first 48 post-operative hours. While there is limited data to support the notion that novel biomarkers have the ability to discriminate ATN from PRA [14,15,30]; our data does not corroborate this, but must be interpreted in light of the lack of consensus amongst the adjudicators. In the past it has been argued that elevation of urinary markers of renal tubular injury with concomitant increased in serum creatinine would indicate true ATN; however, given our inter-rater variability, we could not verify this [17]. Importantly, some of these past studies used specific time parameters to differentiate PRA from ATN while our adjudicators where free to determine a diagnosis regardless of AKI duration. However, despite this difference our ATN cases were of a significantly greater duration compared to PRA. While biomarkers were not included in our adjudication process, we endorse the idea that future research should investigate the utility of biomarkers in distinguishing ATN from other types of kidney injury in a variety of clinical settings.

As nephrologists advance AKI clinical research and re-engage the idea of prophylactic and therapeutic interventional trials we need to 1) recognize that while cardiac surgery produces a reliable rate of AKI, not all elevations in serum creatinine are pathophysiologically equivalent and 2) develop a reliable and consistent system to adjudicate AKI patient diagnoses. It would be unfounded to assume that individuals with PRA have the same risks (short and long term) as those with ATN; yet the published literature often lumps these two clinical entities together to encompass changes in serum creatinine [5,6]. For decades cardiologists, intensivists and oncologists have refined their ability to define patient diagnoses, yet to date there has been little movement on this issue in the field of AKI. More recently clinical adjudication has begun to be relevant and appeared in the AKI literature [10,14,15,30]. However, in these few cases the AKI in question was diverse and the adjudication sought to identify those subjects with PRA in order to determine how well novel biomarkers correlated with AKI outcomes. Additionally, these studies measured biomarkers at the time of clinical AKI or time of peak serum creatinine and our study measured biomarkers in the early post-operative period which may help explain the lack of association in our study.

Our study was limited in that we could only adjudicate 67 cases of AKI. Additionally, our adjudication process was limited by the lack of urinalysis microscopy data. We recommend that future studies should attempt to include some element of urine sediment analysis as microscopy reporting is increasingly standardized and protocol driven allowing for inter-study comparison [31-33]. However, microscopy remains a user dependent tool that displays a tremendous amount of inter-physician variability [34]. Wald and colleagues demonstrated that despite the importance of urinalysis in the diagnosis of renal injury, nephrologists generally achieve only fair to moderate levels of agreement in the identification of important structures on urinalysis and that agreement between nephrologists was not enhanced based on their level of experience. Thus the inclusion of microscopy may have only further muddied our adjudication process given that the pathognomonic “muddy brown casts” are not always present at the time of microscopy even in cases of a known diagnosis of ATN [19]. Additionally, had microscopy been included in the early post-operative period (e.g. ICU arrival), it’s utility remains unclear as there is no published data on the ability of urine microscopy to predict patient outcomes following adult cardiac surgery.

For the past decade several other branches of medicine have succeeded in formalizing the process of patient event adjudication and utilized the results to help further medical research. Over the next decade, adjudication of AKI events will be crucial during the data collection and event analysis of both interventional and prophylactic AKI trials. Our study demonstrates that while adjudication is technically feasible, the interpretation of clinical data in the setting of cardiac surgery remains a challenge. Our experiences, while not perfect, will hopefully provide the building blocks of a formalized process that will allow for inter-study comparison of AKI events. Once a standardized format for adjudication is in place, the role of novel biomarkers in this process can be explored but, currently, such a role remains incipient. Further work is needed to ensure that in the setting of AKI following cardiac surgery, one nephrologist’s PRA is not another nephrologist’s ATN. Until the capacity to produce unified descriptions/definitions is in place, we are instead left with a definition of cardiac surgery associated ATN that is extremely subjective.

## Conclusions

In this nested prospective cohort sub-study of the TRIBE AKI study 56% of those with AKI were adjudicated to have ATN (by at least 2 of the 3 reviewers). Demonstrating that over one-third of patients with AKI may not derive a benefit from interventions aimed at treating or repair tubular injury. Our experiences have led us to conclude that the etiology of AKI after cardiac surgery is multi-factorial and pure AKI etiologies, such as ATN and PRA may not exist. In our study biomarkers did not correlate with the adjudicated etiology of AKI; however the lack of agreement among the adjudicators impacted these results. Further investigation and formalization of the AKI adjudication process, and its interaction with AKI biomarkers, is needed in order to ensure equality in AKI etiologies in future critical care nephrology clinical trials.

## Competing interests

Drs. Parikh and Edelstein are named co-inventor on the IL-18 patent (issued to the University of Colorado).

Dr. Devarajan is named on NGAL patents.

Drs. Coca, Parikh and Devarajan have all served as consultants to Abbott Diagnostics.

Dr. Devarajan is a consultant to Biosite Inc.

Dr. Koyner is a consultant for Astute Medical.

All other authors have no competing interests.

## Authors’ contributions

JLK –conducted the study (patient enrollment/data collection), performed data analysis and interpretation and wrote the manuscript. AXG- conducted the study (patient enrollment/data collection), provided intellectual critique and meaningful revisions to the manuscript. HT- performed data analysis and interpretation and provided meaningful revisions to the manuscript. SGC – Provided Blinded Adjudication of the AKI Cases. LGC - Provided Blinded Adjudication of the AKI Cases. AP- Provided Blinded Adjudication of the AKI Cases. CSP– conducted the study (patient enrollment/data collection), and provided intellectual critique and meaningful revisions to the manuscript. KH – performed data analysis. CRP – conceived, conducted and secured funding for the study performed data analysis and interpretation and wrote the manuscript. All authors read and approved the final manuscript

## Pre-publication history

The pre-publication history for this paper can be accessed here:

http://www.biomedcentral.com/1471-2369/15/105/prepub

## Supplementary Material

Additional file 1 **Strobe Statement—Checklist of items that should be included in reports of *cohort studies.***
Click here for file
